# Using the Log Mean Temperature Difference (LMTD) and ε-NTU Methods to Analyze Heat and Mass Transfer in Direct Contact Membrane Distillation

**DOI:** 10.3390/membranes13060588

**Published:** 2023-06-07

**Authors:** Mohammed A. Almeshaal, Karim Choubani

**Affiliations:** 1 Department of Mechanical Engineering, College of Engineering, Imam Mohammad Ibn Saud Islamic University, Riyadh 11432, Saudi Arabia; 2 Research Unit: Mechanical Modeling, Energy & Materials (M2 EM), UR17ES47, National School of Engineers of Gabes (ENIG), Avenue of Omar Ib-Elkhattab, Zrig 6023, Gabes, Tunisia

**Keywords:** direct contact membrane distillation, heat exchanger, log mean temperature difference, effectiveness-NTU method, flux prediction

## Abstract

In direct contact membrane distillation (DCMD), heat and mass transfers occur through the porous membrane. Any model developed for the DCMD process should therefore be able to describe the mass transport mechanism through the membrane, the temperature and concentration effects on the surface of the membrane, the permeate flux, and the selectivity of the membrane. In the present study, we developed a predictive mathematical model based on a counter flow heat exchanger analogy for the DCMD process. Two methods were used to analyze the water permeate flux across one hydrophobic membrane layer, namely the log mean temperature difference (LMTD) and the effectiveness-NTU methods. The set of equations was derived in a manner analogous to that employed for heat exchanger systems. The obtained results showed that the permeate flux increases by a factor of approximately 220% when increasing the log mean temperature difference by a factor of 80% or increasing the number of transfer units by a factor of 3%. A good level of agreement between this theoretical model and the experimental data at various feed temperatures confirmed that the model accurately predicts the permeate flux values for the DCMD process.

## 1. Introduction

A scarcity of potable water presents a serious problem in many countries. Due to the ample water resources in seas and oceans, desalination is becoming an increasingly attractive solution. Studies have demonstrated the potential of membrane distillation for direct water desalination, and the simplest process for membrane distillation is direct contact membrane distillation.

In direct contact membrane distillation (DCMD), a difference in partial pressure through a membrane is generated through a temperature difference between feeds of hot and cold liquids on both sides of the hydrophobic membrane. The volatile molecules evaporate at the liquid/steam transition, pass through the membrane and condense at the steam/liquid transition.

Mohsen et al. [1] used computational fluid dynamics (CFD) to simulate the direct contact membrane distillation (DCMD) process. They showed that the pressure gradient of water vapor is increased when cold fluid flows in the permeate channel because the temperature increases along the membrane contactor. Janajreh et al. [2], meanwhile, developed a numerical model for a DCMD system, with them considering numerous parameters in an attempt to achieve an optimal condition. The results show that when mass flux is the objective, one should use a higher temperature, thicker membrane, a relatively lower conductivity, and a higher velocity in counter and converging flow design.

Swaminathan et al. [3] developed a simplified numerical model for membrane distillation energy-efficiency modules based on the heat exchanger analogy. It was found that, over a wide range of operating conditions, the results of the simplified heat exchanger model were within 11% of the results from more detailed simulations. Karam et al. [4] presented a predictive dynamic model for direct contact membrane distillation and discussed the results under numerous dynamic parameters, with them showing that the temperature distribution profiles along the length of the module were nonlinear and that the behavior of the temperature polarization coefficient was asymptotic at high inlet velocities.

A. Khalifa et al. [5] used an analytical model based on heat and mass transfer equations to predict the system performance at different parameters. They showed that the permeate flux increases with increasing feed temperature, permeate flow rate, feed flow rate, and pore size, and it decreases along with increasing feed concentration and permeate temperature. Park et al. [6] developed a two-dimensional CFD model to investigate the performance of a direct contact membrane distillation system under different conditions. Their numerical simulation showed that the permeate flux increased along with the inlet feed temperature, with it also verifying that the feed temperature had a greater impact on the water flux than the permeate temperature.

Soukane et al. [7] implemented a three-dimensional CFD model to predict heat and mass transfer in a DCMD module, with their simulation results showing that momentum and heat transport strongly affected the distribution of salinity and permeate fluxes over the membrane surface, while the temperature and concentration polarization follows the flow pattern distribution. In addition, at low operating temperatures, dead zones tend to accumulate salt. Next, Lee et al. [8] used an experimentally validated model to simulate the effect of convection heat and mass transfer on the MD performance parameters. They investigated the mean permeate flux, temperature polarization coefficient, and specific energy consumption in a direct contact membrane distillation process. Their results showed that, with low mass transfer coefficients, the temperature polarization coefficient is high, while the effect of the convection heat transfer coefficient on the process performance was not significant. Moreover, a rise in the mass transfer coefficient increased the effect of the convection heat transfer coefficient on water production.

Long et al. [9] proposed a modified model for characterizing the heat and mass transfer in the DCMD process in order to evaluate the effect of heat recovery, the gain output ratio, and the mass recovery rate on the performance of a DCMD system. The obtained results show that the gain output ratio reaches a maximum value for the optimal mass flow rate, while the mass recovery rate reaches a maximum at higher flow rates where the gain output ratio decreases. Perfilov et al. [10] developed a predictive model for DCMD based on describing the momentum, mass, and heat balances through systems of ordinary differential, partial differential, and algebraic equations. The obtained results effectively estimated the effects of the operating conditions and physical membrane properties on the performance of DCMD, such as the velocity, concentration, and temperature distributions in the DCMD units.

Kuang et al. [11] developed a numerical simulation of the DCMD process based on computational fluid dynamics, with their results revealing that making a structural modification by employing baffles can enhance the temperature polarization phenomenon and decrease the concentration polarization phenomenon, thus increasing the water flux production. Lou et al. [12] developed and experimentally validated a two-dimensional CFD code to simulate heat and mass transport in a DCMD system, with this showing that vapor flux, temperature, and concentration vary significantly in the downstream direction, such that they could not be accurately predicted by common Nusselt and Sherwood correlations. Moreover, temperature polarization considerably decreases with the inlet flow rate, whereas concentration polarization decreases only slightly.

Kim et al. [13] proposed a mathematical model that was confirmed through experimental measurements for predicting membrane tortuosity based on measured porosity to calculate the operational parameters in DCMD systems, particularly for water flux production. It was found that the differences in water flux predicted by the proposed tortuosity model increased considerably as the width and length of the membrane were increased. Lou et al. [14], meanwhile, developed the CFD code to simulate unsteady two-dimensional heat and mass transport in DCMD systems with cylindrical spacers. The results showed that although unsteady vortex structures are able to mix temperature polarization layers with the bulk, they are unable to mix the concentration layers. Moreover, spacers often increase the permeate flux at the expense of greater mineral scaling.

Lim et al. [15] used a lab-scale DCMD system to investigate the effect of varying the concentration of organic and inorganic contents in feed bulk on wetting membrane pores. Their results show that a salt concentration rise in the added solution increased the membrane wetting. Moreover, salts could pass through wetted pores and form a layer of scale on the distillate side of the membrane. Noamani et al. [16] developed a theoretical model that was based on heat and mass transfer and simulated by the ε-NTU method. The obtained results showed that the feed temperature and physical membrane characteristics were the most influential parameters on water production and energy efficiency in the DCMD system. Moreover, the developed model was also used to determine the optimum conditions for achieving greater performance in terms of permeate flux and energy efficiency.

This present study was inspired by the log mean temperature difference (LMTD) and the effectiveness-NTU methods, so a theoretical model was developed based on a heat and mass transfer analysis of the DCMD process. The developed model was also used to analyze the performance of the process in terms of permeate flux.

## 2. Modeling and Configuration Description

**Figure 1:**
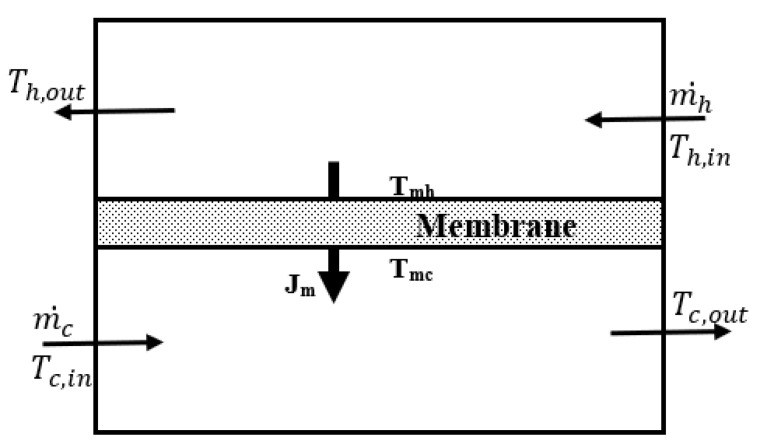
Permeate flux across a single-layer hydrophobic membrane in the DCMD process.

For the current modeling, the system is defined in Figure 1. This consists of a hot-water channel and a cold-water channel separated by a porous hydrophobic membrane material. The hot-water temperature drops over the feed side to the membrane surface temperature, while the cold-water temperature rises across the cold layer to the membrane surface temperature as the vapor condenses into the fresh water. The driving force is therefore the vapor pressure difference between the hot and cold membrane surfaces.

In an analysis of the DCMD process for water desalination, it is convenient to combine all the various thermal resistances to heat flow from hot water into cold water into a single resistance R and express the rate of heat transfer between the two fluids as:

(1) $$Q_{\mathrm{tot}}=\;U.A.\Delta T$$

(2) $$U=1/(1/h_{h}+\delta /K_{m}+1/h_{c})$$

According to the first law of thermodynamics, we have:

(3) $$Q_{\mathrm{tot}}=\dot{m_{c}}C_{\mathrm{pc}}\left(T_{c,\mathrm{out}}-{\;T}_{c,\mathrm{in}}\right)$$

(4) $$Q_{\mathrm{tot}}=\dot{m_{h}}C_{\mathrm{ph}}\left(T_{h,\mathrm{in}}-{\;T}_{h,\mathrm{out}}\right)$$

Next, we define the heat capacity rate for the hot- and cold-water flows as:

(5) $$C_{c}=\dot{m_{c}}C_{\mathrm{pc}}$$

(6) $$C_{h}=\dot{m_{h}}C_{\mathrm{ph}}$$

With the definition of the heat capacity rate given above, Equations (3) and (4) can also be expressed as:

(7) $$Q_{\mathrm{tot}}={\;C}_{c}\left(T_{c,\mathrm{out}}-{\;T}_{c,\mathrm{in}}\right)$$

(8) $$Q_{\mathrm{tot}}={\;C}_{h}\left(T_{h,\mathrm{in}}-{\;T}_{h,\mathrm{out}}\right)$$

### 2.1. The Heat Transfer Model

#### 2.1.1. The Log Mean Temperature Difference (LMTD) Method

In the DCMD process, the temperature difference between the hot and cold water varies along the membrane, so it is convenient to use a mean temperature difference $\Delta T_{\mathrm{lm}}$ in the equation:

(9) $$Q_{\mathrm{tot}}=\;U.A.\Delta T_{\mathrm{lm}}$$

In order to develop a formula for the equivalent average temperature difference between the hot and cold water, we consider the counter flow configuration:

(10) $$\Delta T_{\mathrm{lm}}=\frac{\Delta T_{1}-\Delta T_{2}}{\ln\left(\frac{\Delta T_{1}}{\Delta T_{2}}\right)}$$

This difference, called the log mean temperature difference, is a form of average temperature difference that is suitable for use in the analysis of heat exchangers. Here, $\Delta T_{1}$ and $\Delta T_{2}$ represent the temperature difference between the hot and cold water.

In the case of counter flow:

(11) $$\Delta T_{1}={\;T}_{h,\mathrm{in}}-{\;T}_{c,\mathrm{out}}$$

(12) $$\Delta T_{2}={\;T}_{h,\mathrm{out}}-{\;T}_{c,\mathrm{in}}$$

The LMTD method is easy to use in the DCMD process analysis when the inlet and outlet temperatures of the hot and cold fluids are known or can be determined from the energy balance. Once $\Delta T_{\mathrm{lm}}$***,*** the mass flow rates, and the overall heat-transfer coefficient are available, the heat transfer surface area of the membrane can be determined from Equation (9).

When the mass flow rates and inlet and outlet temperatures of the hot and cold fluids are specified, the LMTD method is very suitable for determining the size of the membrane needed to realize prescribed outlet temperatures.

In this study, we applied this method to calculate the outlet temperatures of hot and cold fluids and the heat transfer rate for prescribed fluid mass flow rates and inlet temperatures when the size and the type of the membrane were specified. The heat transfer surface area A of the membrane is known in this case; however, the outlet temperatures are not, so the objective here was to determine the heat transfer performance of a specified membrane and thus determine whether that membrane will perform appropriately.

#### 2.1.2. The Effectiveness-NTU Method

The LMTD method could also be used for the alternative problem of determining the performance of the DCMD process, but this procedure would require monotonous iterations, making it impractical. In an attempt to eliminate such an iteration from the solution of this problem, and thereby simplify the analysis, the effectiveness-NTU (ε-NTU) method was applied.

This method is based on the heat transfer effectiveness (ε)*,* which is a dimensionless parameter, defined as follows:

(13) $$\varepsilon =\frac{Q_{\mathrm{tot}}}{Q_{\max}}=\frac{\mathrm{Actual}\;\mathrm{heat}\;\mathrm{transfer}\;\mathrm{rate}}{\mathrm{Maximum}\;\mathrm{possible}\;\mathrm{heat}\;\mathrm{transfer}\;\mathrm{rate}}$$

From the energy balance on the hot and cold fluids, the actual heat transfer rate can be expressed as:

(14) $$Q_{\mathrm{tot}}={\;C}_{c}\left(T_{c,\mathrm{out}}-{\;T}_{c,\mathrm{in}}\right)={\;C}_{h}\left(T_{h,\mathrm{in}}-{\;T}_{h,\mathrm{out}}\right)\;$$

To determine the maximum possible heat transfer rate, we first posit that the maximum temperature difference as the difference between the inlet temperatures of the hot and cold water:

(15) $$\Delta T_{\max}={\;T}_{h,\mathrm{in}}-{\;T}_{c,\mathrm{in}}$$

When $C_{c}\;\neq {\;C}_{h}$, which is usually the case, the water with the lower heat capacity rate will experience a larger temperature change, so it will be the first to reach a maximum temperature, at which point any heat transfer will come to a halt. Thus, the maximum possible heat transfer rate is:

(16) $$Q_{\max}={\;C}_{\min}\;\left(T_{h,\mathrm{in}}-{\;T}_{c,\mathrm{in}}\right)$$

where $C_{\min}$ is the least of $C_{c}=\dot{m_{c}}C_{\mathrm{pc}}$ and $C_{h}=\dot{m_{h}}C_{\mathrm{ph}}$.

Determining $Q_{\max}$ requires the inlet temperature of the hot and cold water and their mass flow rates to be available, and fortunately, these are usually specified. Thus, once the effectiveness of the membrane is known, the actual heat transfer rate $Q_{\mathrm{tot}}$ can be determined as:

(17) $$Q_{\mathrm{tot}}={\;\varepsilon Q}_{\max}={\;\varepsilon C}_{\min}\;\left(T_{h,\mathrm{in}}-{\;T}_{c,\mathrm{in}}\right)$$

The effectiveness therefore enables us to determine the heat transfer rate without knowing the outlet temperatures of the water flows.

Effectiveness relations typically involve the dimensionless group $\frac{\mathrm{UA}}{C_{\min}}$. This quantity is called the number of transfer units (NTU), which is expressed as:

(18) $$\mathrm{NTU}=\frac{\mathrm{UA}}{C_{\min}}$$

where A is the heat transfer surface area and U is the overall heat transfer coefficient, which is defined as:

(19) $$U=\frac{1}{\frac{1}{h_{h}}+\frac{\delta }{K_{m}}+\frac{1}{h_{c}}}$$

The value of NTU is a measure of the heat transfer surface area A. In heat analysis, it is also convenient to use another dimensionless quantity called the capacity ratio C, which is defined as:

(20) $$C=\frac{C_{\min}}{C_{\max}}$$

It can be shown that the effectiveness is a function of the number of transfer units (NTU) and the capacity ratio C:

(21) $$\varepsilon =\mathrm{function}\;\left(\mathrm{NTU},\;C\right)$$

An extensive range of effectiveness charts and relations are available in the literature.

During a phase-change process, all effectiveness relations reduce to [17]:

(22) $$\varepsilon ={\;\varepsilon }_{\max}=1-\exp\left(-\mathrm{NTU}\right)$$

### 2.2. Mass Transfer Model

#### 2.2.1. Relevant Quantities and Relations Involved in Mass Transfer Modeling

The mass flux $J_{m}$ depends on the diffusion coefficient $B_{m}$ as well as on the difference in partial vapor pressure between the hot and cold water on both sides of the membrane $\left(P_{\mathrm{mh}}-{\;P}_{\mathrm{mc}}\right)$. It is given by the following equation:

(23) $$J_{m}={\;B}_{m}\;\left(P_{\mathrm{mh}}-{\;P}_{\mathrm{mc}}\;\right)$$

For pure water,${\;P}_{\mathrm{mh}},{\;P}_{\mathrm{mc}}$ are determined by Antoine’s equation, which relates these pressures to the temperatures of the membrane surfaces as follows:

(24) $$P_{\mathrm{mh}}=\exp\left[\left(23.1964\right)-\frac{3816.44}{T_{\mathrm{mh}}-46.13}\right]$$

(25) $$P_{\mathrm{mc}}=\exp\left[\left(23.1964\right)-\frac{3816.44}{T_{\mathrm{mc}}-46.13}\right]$$

where $T_{\mathrm{mh}}$ and $T_{\mathrm{mc}}$ are the average membrane surface temperature on the hot-water side and the cold-water side, respectively. $T_{\mathrm{mh}}$ and $T_{\mathrm{mc}}$ are given by the following equations:

(26) $$T_{\mathrm{mh}}=T_{h,\mathrm{in}}-\frac{Q_{\mathrm{tot}}}{h_{h}}$$

(27) $$T_{\mathrm{mc}}=T_{c,\mathrm{in}}+\frac{Q_{\mathrm{tot}}}{h_{c}}$$

The convective heat transfer coefficient $h_{i}$ is given by the following equation:

(28) $$h_{i}=\frac{\mathrm{Nu}\times K_{i}}{d_{h,i}}$$

where i = h (hot) or c (cold).

The Nusselt number is given as a function of the Prandtl number and the Reynolds number (Table 1), where the Prandtl number Pr is given as follows:

(29) $$\mathrm{Pr}=\frac{\mu_{i}.C_{p,i}}{K_{i}}$$

While the Reynolds number is defined as follows:

(30) Re=ρ.vi.dh,iμi

**Table 1:** Numerical correlations used to calculate the Nusselt number.

Correlations	Flow Regime	References
Nu=1.86Re.PrLdh13 (31)	Laminar	[18]
Nu=0.0231+6dhLRe0.8Pr13 (32)	Turbulent	[19]

#### 2.2.2. Diffusion Coefficient of the Membrane ($B_{m}$)

Theoretical and experimental studies have shown that the combined Knudsen-molecular diffusion is the most efficient approach that best reflects reality, so we adopted this mode of diffusion for our study [20]. Under this approach, the coefficient $B_{m}$ is given by the following expression:

(33) $$B_{m}={\left[\frac{3.\tau .\delta }{\varepsilon_{0}.d_{p}}\;\sqrt{\frac{\pi .R.T_{\mathrm{mav}}}{8.M_{w}}}+\frac{\tau .\delta .P_{a}.R.T_{\mathrm{mav}}}{\varepsilon_{0}.\mathrm{PD}.M_{w}}\right]}^{-1}$$

where $T_{\mathrm{mav}}=\frac{T_{\mathrm{mh}}+{\;T}_{\mathrm{mc}}}{2}$ is the absolute mean temperature in the pores.

The diffusivity of water vapors (P.D) through the static air in the membrane is calculated using the following expression [16]:

(34) $$P.D=1.895\times 10^{-5}\times T_{\mathrm{mav}}^{2.072}\;$$

## 3. Numerical Simulation

To predict the permeate flux production, some code was established and implemented in MATLAB. An iterative approach was carried out by simultaneously solving the heat and mass transfer equations to calculate the surface temperatures of the membrane before deducing the permeate flux.

**Figure 2:**
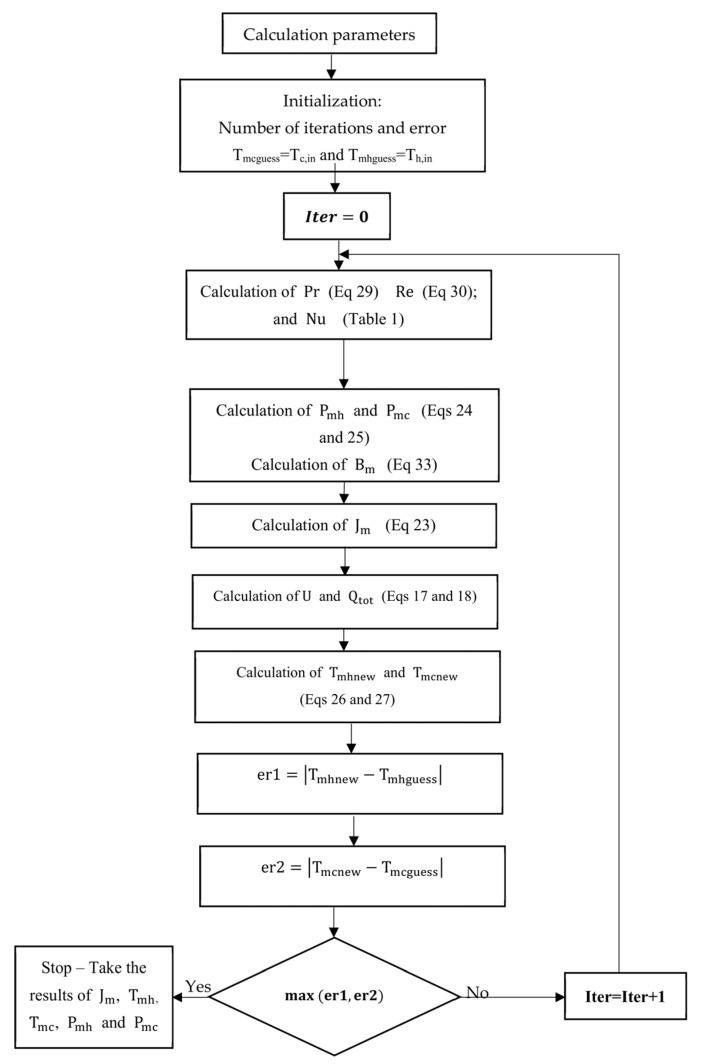
Flowchart for the calculation process.

Initially, the temperatures of the hot and cold surfaces of the membrane were estimated to be equal to the inlet temperatures of the hot and cold water (i.e., $T_{\mathrm{mh}0}={\;T}_{h,\mathrm{in}0}$ and $T_{\mathrm{mc}0}={\;T}_{c,\mathrm{in}0}$). These temperature values were used to calculate the vapor pressures $P_{\mathrm{mh}}$ and $P_{\mathrm{mc}}$ and then estimate the permeate flow $J_{m}$. The ($T_{\mathrm{mh}}$) value was then decreased by $\frac{Q_{\mathrm{tot}}}{h_{h}}$, while the ($T_{\mathrm{mc}}$) value was increased by $\frac{Q_{\mathrm{tot}}}{h_{c}}$. The new values of $T_{\mathrm{mh}}$ and $T_{\mathrm{mc}}$ were then used to estimate a new value for the permeate flux $J_{m}$. The above procedures were repeated until the maximum difference between two consecutive values for the temperatures $T_{\mathrm{mh}}$ and $T_{\mathrm{mc}}$ was within an error margin of 0.1%. The flowchart in Figure 2 depicts the multistep procedure that was adopted to predict the permeate flux:

## 4. Results and Discussion

In this work, the influences of the feed solutions’ temperature and the membrane characteristics on the permeate flux for a pure water solution were simulated and analyzed using the LMTD and ε−NTU methods.

### 4.1. Model Validation

In this section, we first validate the predictions of the developed mathematical model against the experimental data obtained by Andrjesdóttir et al. [21]. In our code, we used the same membrane properties and geometrical constants as Andrjesdóttir et al. [21] in their experiments (Table 2).

**Figure 3:**
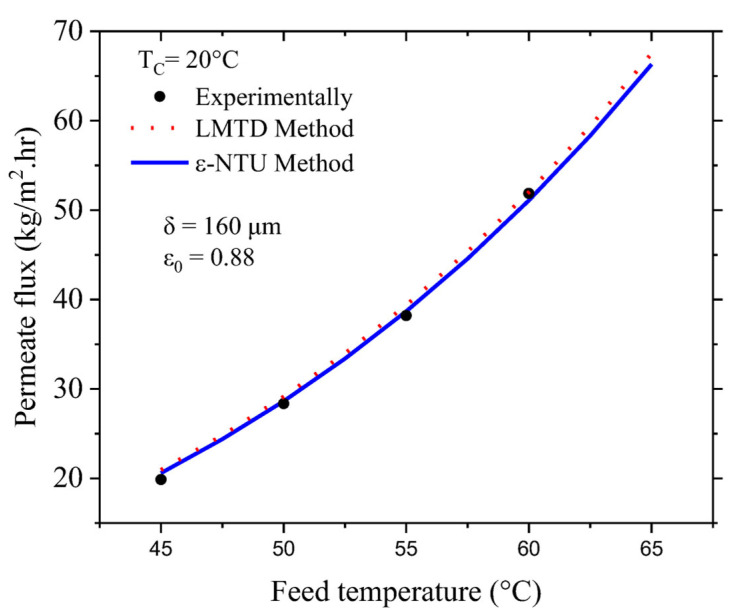
Results for the validation of the model with Andrjesdóttir et al. [21].

This validation procedure was performed for feed water temperatures varying from 45 °C to 65 °C, a constant permeate water temperature equal to 20 °C, a hot-water flow of 12 L/min., and a cold-water flow of 4 L/min. Figure 3 shows the corresponding goodness of fit between the experimental results (the black points) of Andrjesdottir et al. [21] and our theoretical results using the LMTD method (the red points) and the ε-NTU method (the blue line). The relative error between the experimental and theoretical results is within 4%, with the results of the ε-NTU method being closer to the experimental results. From Figure 3, we can see that a 20 °C rise in the feed temperature leads to a 70% increase in the vapor flux.

**Figure 4:**
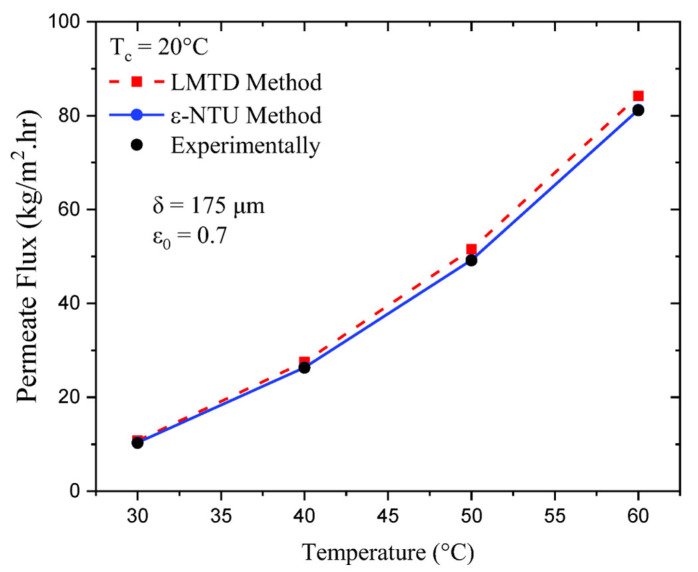
Results for the validation of the model with Cath et al. [22].

To support our developed code, the predicted permeate flux was validated against the experimental work of Cath et al. [22]. This validation was performed for feed water temperatures varying from 30 °C to 60 °C, a constant permeate water temperature equal to 20 °C, and feed and permeate velocities equal to 1.75 m/s. Figure 4, which depicts the effects of increasing the feed temperature, shows that there is good agreement between the model predictions and experimental results with a relative error of less than 5%.

### 4.2. Effect of Log Mean Temperature on the Permeate Flux

**Figure 5:**
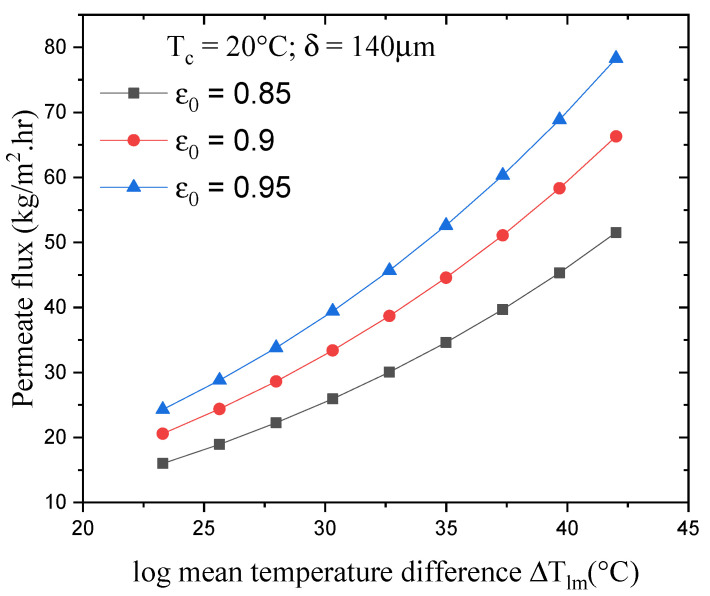
Effect of the log mean temperature difference on the permeate flux for different membrane porosities.

Figure 5 illustrates the effect of the log mean temperature $\Delta T_{\mathrm{lm}}$ on the DCMD flux for a variable membrane porosity and constant permeate temperature T_c,in_ and membrane thickness δ. Increasing $\Delta T_{\mathrm{lm}}$ enhances the permeate flux significantly. This can be attributed to an increase in the feed-side membrane surface temperature (T_h,in_), which in turn increases the driving force and partial vapor pressure gradient. Our results show that, with constant membrane porosity, an 80% increase in $\Delta T_{\mathrm{lm}}$ increases the permeate flux by approximately 222%. Increasing the membrane porosity, meanwhile, increases the permeability of the membrane, decreases the heat loss effect through conduction, and elevates the permeate flux [23]. The reason behind lower heat loss is the presence of air or water vapor in the pores. Therefore, as shown in Figure 5, increasing the membrane porosity from 0.85 to 0.95 μm increased the permeate flux by 52%, with it going from 51.5 to 78.3 kg/m^2^ h (for the highest value of $\Delta T_{\mathrm{lm}}$).

**Figure 6:**
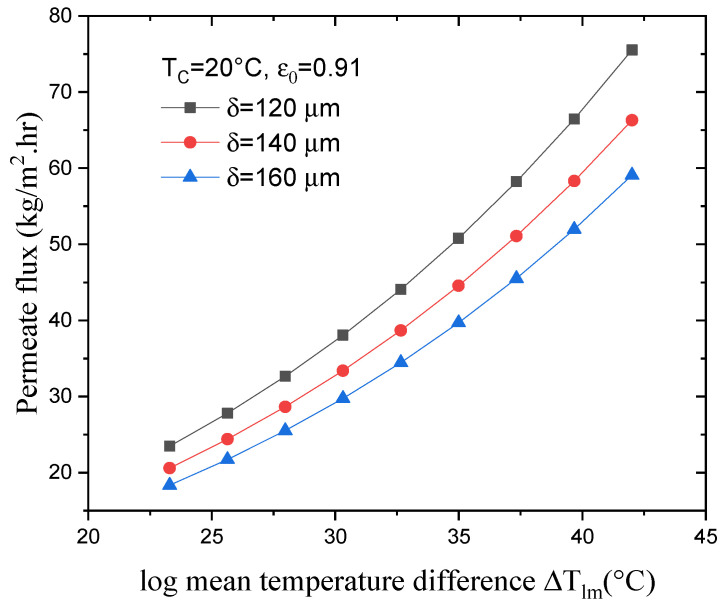
Effect of log mean temperature difference on permeate flux with various membrane thicknesses.

Figure 6 illustrates the effect of varying $\Delta T_{\mathrm{lm}}$ on the DCMD flux for variable membrane thicknesses with a constant permeate temperature T_c,in_ and membrane porosity  ε. The results show that, with a constant membrane thickness, an 80% increase in $\Delta T_{\mathrm{lm}}$ leads to an increase in the permeate flux of approximately 220%. Increasing the membrane thickness, meanwhile, leads to greater resistance against the mass and heat transfer, resulting in a higher membrane surface temperature on the feed side (T_mhin_) and a lower total mass transfer coefficient. There is an inverse relationship between the permeate flux and thickness of the membrane. As the thickness increases, the flux decreases. The thicker the membrane, the higher the mass transfer resistance is, resulting in reduced permeate flux [24]. Therefore, as illustrated in Figure 6, increasing the membrane thickness from 120 to 160 μm decreased the permeate flux by 28%, from 75.5 to 59 kg/m^2^.h (for the highest value of $\Delta T_{\mathrm{lm}}$).

### 4.3. Effect of the Number of Transfer Units (NTU) on the Permeate Flux

**Figure 7:**
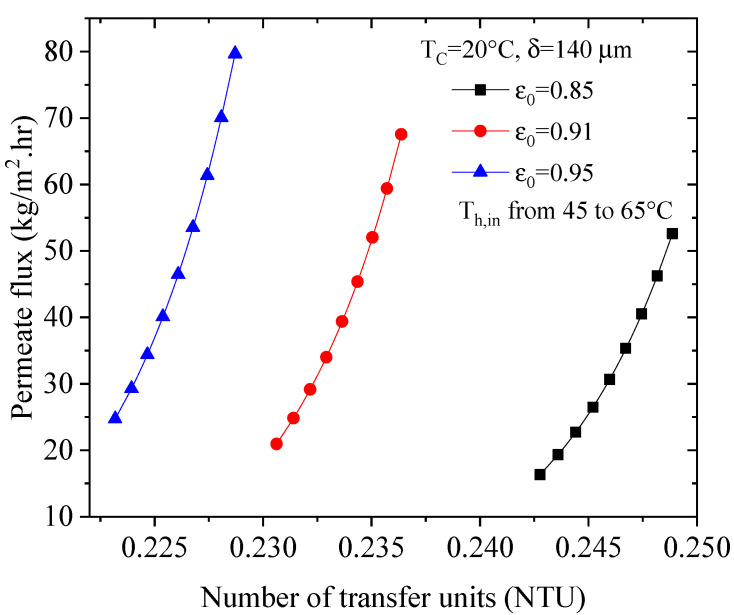
Effect of increasing NTU on the permeate flux with various porosities.

In ε-NTU heat transfer analysis, it is traditional to present the results as plots of efficiency (ε) versus the number of transfer units (NTU), but for the DCMD module performance, it is more appropriate to present the output water production rate versus NTU because the former is the parameter of concern. The effect of NTU on DCMD performance, with various membrane porosities, is illustrated in Figure 7. Upon increasing the NTU from 0.24441 to 0.24887 (an increase of about 2.5%), the permeate flux increases by 222%. A possible explanation for this is an increase in the overall heat transfer coefficient due to an increase in the convection heat transfer coefficients, which in turn leads to a greater driving force and permeate flux.

**Figure 8:**
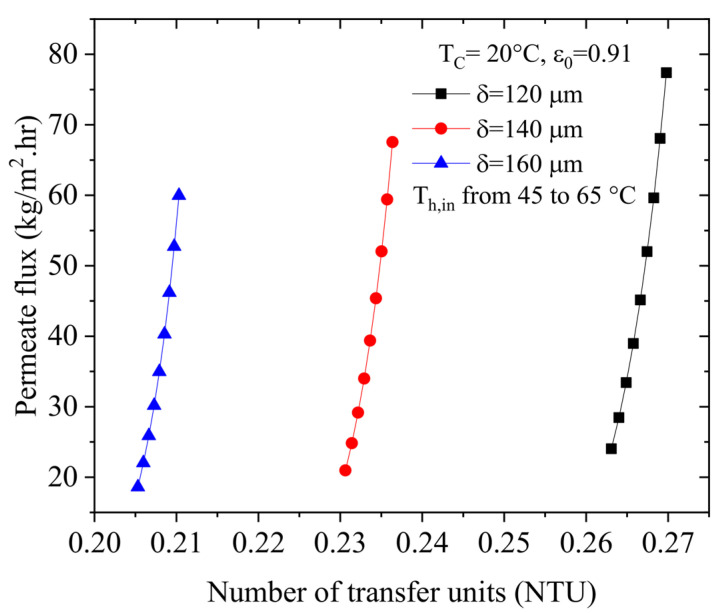
Effect of increasing NTU on the permeate flux with various membrane thicknesses.

Next, Figure 8 illustrates the effect of increasing NTU on the permeate flux for various membrane thicknesses. As can be seen, a 2.5% increase in the NTU value leads to an increase of 222% in the water flux.

### 4.4. Effect of Feed Temperature on the NTU

**Figure 9:**
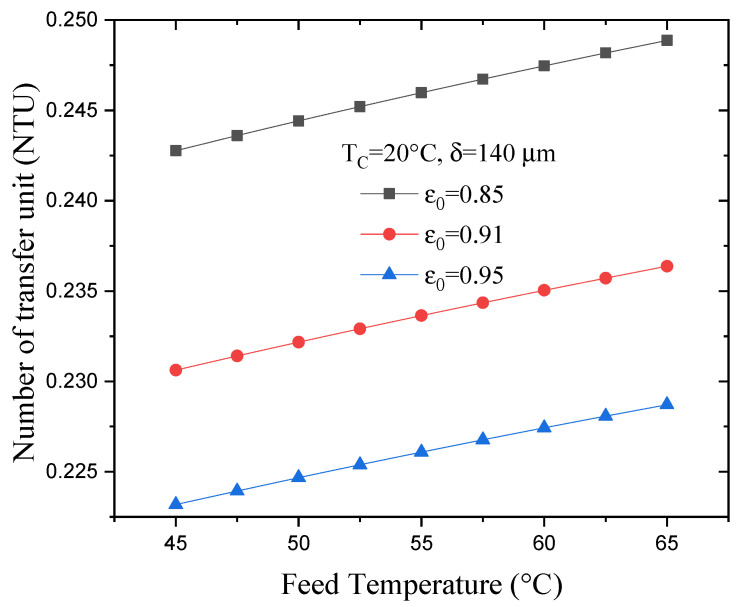
Effect of varying feed temperature on the NTU with various porosities.

Figure 9 illustrates the effect of varying the feed temperature on the NTU for various porosities but with a constant membrane thickness and permeate temperature. As can be seen, increasing the hot-water temperature from 45 °C to 65 °C causes the NTU to increase by 2.5%. Moreover, an increase in the membrane porosity reduces the NTU, such that increasing the membrane porosity from 0.85 to 0.95—with the feed temperature remaining constant—caused the NTU to decrease by 9%. This is possibly because an increase in membrane porosity leads to a decrease in the heat transfer surface area.

**Figure 10:**
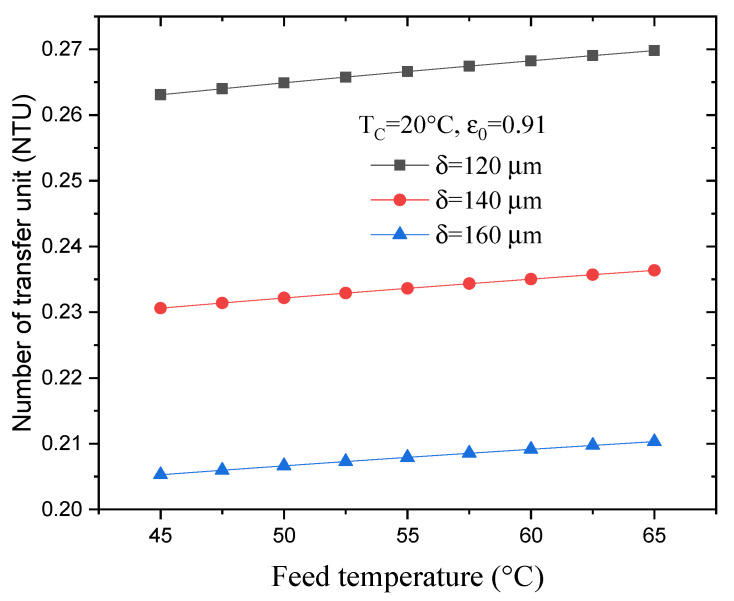
Effect of varying feed temperature on the NTU for various thicknesses.

Figure 10 shows the effect of varying the feed temperature on the NTU for various membrane thicknesses, but with a constant membrane porosity and permeate temperature. As can be seen, increasing the feed temperature from 45 °C to 65 °C causes the NTU to increase by 2.5%, but increasing the membrane thickness from 120 to 160 μm reduces the NTU by about 28%. This is likely the result of a decrease in the overall heat transfer coefficient due to a decrease in the conduction heat transfer coefficients.

## 5. Conclusions

This study has drawn some parallels between the classical method for heat transfer and the LMTD and ε-NTU methods for membrane distillation. It has shown that even with changes in membrane porosity, membrane thickness, and feed and permeate temperatures, the LMTD and ε-NTU methods provide good estimations that are comparable to those from the classical model. The difference in the results between the two methods is also negligible because the membrane area is constant. In summary, among all the studied parameters, the NTU was found to have the most significant effect on permeate flux, followed by the LMTD. In terms of membrane properties, membrane thickness and porosity were found to have a great effect on the permeate flux.

## Figures and Tables

**Table 2:** Membrane characteristics and geometrical constants used in the validation process [21,22].

Symbols	Values Used in Both Our Code and the Work of Andrjesdóttir et al. [21]	Values Used in Both Our Code and the Work of Cath et al. [22]
Δ	160 μm	175 μm
$\varepsilon_{0}$	0.88	0.7
$K_{g}$	0.029 W/mK	0.029 W/mK
$K_{P}$	0.259 W/mK	0.259 W/mK
$d_{p}$	0.2 μm	0.45 μm
A	$11.7\times 10^{-3}{\;m}^{2}$	$6\times 10^{-4}{\;m}^{2}$
$d_{h}$	$5.2\times 10^{-3}\;m$	$2.4\times 10^{-3}\;m$

## Nomenclature

B_m_	Mass transfer coefficient (kg × m^−2^ × h^−1^ × Pa^−1^)
C_p_	Specific heat of water (J × kg^−1^ × K^−1^)
C_c_	Heat capacity rate for the cold water (W × K^−1^)
C_h_	Heat capacity rate for the hot water (W × K^−1^)
C_min_	Min ($\dot{m_{c}}C_{\mathrm{pc}}$, $\dot{m_{h}}C_{\mathrm{ph}}$) (W × K^−1^)
C_max_	Max ($\dot{m_{c}}C_{\mathrm{pc}}$, $\dot{m_{h}}C_{\mathrm{ph}}$) (W × K^−1^)
C	Capacity ratio ($C=\frac{C_{\min}}{C_{\max}}$)
d_h_	Hydraulic diameter (m)
d_p_	Mean pore diameter of the membrane (m)
A	Surface area of the membrane (m^2^)
h_c_	Heat transfer coefficient of permeate (W × m^−2^× K^−1^)
h_h_	Heat transfer coefficient of feed (W × m^−2^ × K^−1^)
h_m_	Heat transfer coefficient of the membrane (W × m^−2^ ×K^−1^)
J_m_	Permeate flux (kg × m^−2^ × h^−1^)
K_i_	Mean thermal conductivity (W × m^−1^ ×K^−1^)
K_m_	Thermal conductivity of the membrane (W × m^−1^ × K^−1^)
K_g_	Thermal conductivity of the gas filling the membrane pores (W × m^−1^ × K^−1^)
K_p_	Thermal conductivity of the polymer (membrane material) (W × m^−1^ × K^−1^)
M_w_	Molecular weight of water (g × mol^−1^)
$\dot{m_{c}}$	Mass flow rate of the cold water (kg × s^−1^)
$\dot{m_{h}}$	Mass flow rate of the hot water (kg × s^−1^)
Nu	Nusselt number (Dimensionless)
Pa	Air pressure inside the membrane pores (Pa)
P×D	Diffusion coefficient in the pores (Pa × m^2^ × s ^−1^)
P_mc_	Membrane interface partial pressure of water molecule on the permeate side (Pa)
P_mh_	Membrane interface partial pressure of water molecule on the feed side (Pa)
Pr	Prandtl number (Dimensionless)
Q_tot_	Rate of heat transfer between hot and cold fluids (W)
Q_max_	Maximum possible heat transfer rate (W)
R	Universal gas constant (8.314472 J × mol^−1^ × K^−1^)
Re	Reynolds number (Dimensionless)
T_c_	Permeate temperature (°C)
T_h_	Feed temperature (°C)
T_mc_	Membrane interface temperature on permeate side (°C)
T_mh_	Membrane interface temperature on feed side (°C)
T_mav_	Mean temperature (T_mav_ = (T_h_ + T_c_)/2) (°C)
U	Overall heat transfer coefficient (W × m^−2^ × K^−1^)
ΔT_lm_	Log mean temperature difference (°C)
ε	Effectiveness
NTU	Number of transfer units
v	Average velocity (m × s^−1^)
δ	Membrane thickness (m)
ε_0_	Membrane porosity (%)
µ	Dynamic viscosity (Pa × s)
ν	Kinematic viscosity (m^2^ × s^−1^)
ρ	Density (Kg × m^−3^)
τ	Membrane tortuosity
Subscripts:	
c	cold
h	hot
m	membrane surface
in	inlet
out	outlet
